# Nutrition Knowledge, Dietary Habits, and Food Labels Use—A Representative Cross-Sectional Survey among Adults in Poland

**DOI:** 10.3390/ijerph191811364

**Published:** 2022-09-09

**Authors:** Adam Żarnowski, Mateusz Jankowski, Mariusz Gujski

**Affiliations:** 1 Department of Public Health, Medical University of Warsaw, 02-097 Warsaw, Poland; 2 School of Public Health, Centre of Postgraduate Medical Education, 01-826 Warsaw, Poland

**Keywords:** nutrition knowledge, front-of-package label, calorie labeling, diet, dietary habits, nutritional awareness, Poland

## Abstract

An unhealthy diet is an important risk factor for disability and premature death. This study aimed to assess nutrition knowledge, dietary habits, and food label use among adults in Poland as well as to identify factors associated with diet-related behaviors. A cross-sectional survey was carried out in July 2020 on a non-probability quota-based sample of 1070 adult citizens of Poland. The most common sources of nutrition knowledge were news websites (41.8%) or family/friends (32.4%). Over one-quarter of adults in Poland were on a diet (28.7%). Over one-tenth of respondents (11.9%) consumed less than three meals per day. Half of the respondents (50.3%) declared that they use food labels when shopping, and 15.4% checked the nutrition information on restaurant menus. Female gender (OR:1.70; 95%CI:1.26–2.29; *p* < 0.001), presence of chronic diseases (OR:1.83; 95%CI:1.37–2.44; *p* < 0.001), regular physical activity (*p* < 0.001), and being a non-smoker (OR:1.45; 95%CI:1.02–2.06; *p* = 0.04) were significantly associated with higher odds of being on a diet. Females (OR:1.63; 95%CI:1.24–2.15; *p* < 0.001), respondents with higher education (OR:1.53; 95%CI:1.17–2.01; *p* = 0.002), those who had never been married (OR:1.49; 95%CI:1.07–2.07; *p* = 0.02), respondents with chronic diseases (OR:1.73; 95%CI:1.30–2.31; *p* < 0.001), those with regular physical activity (*p* < 0.05), as well as non-smokers (OR:1.42; 95%CI:1.04–1.95; *p* = 0.03) had higher odds of checking the food labels. This study showed a significant gap in nutrition knowledge among adults in Poland.

## 1. Introduction

An unhealthy diet is an important risk factor for disability and premature death [1,2]. In 2017, more than 10 million deaths and 250 million disability-adjusted life-years (DALYs) were attributed to dietary risk factors [3]. Every year, millions of people around the world make attempts to change their dietary patterns [4]. The definition of a healthy diet is continually shifting [5]. However, the major principle of a healthy diet is to provide a balance between energy intake (calories) and energy expenditure [5,6]. Adequate consumption of fruit, vegetables, legumes, nuts, and whole grains is considered a key element of a healthy diet [6].

Nutrient recommendations depend on individual characteristics, including gender, age, lifestyle, daily habits, type of occupation, and physical activity level [6]. Cultural and religious factors, availability of food products, and consumer behaviors also shape dietary choices [6,7]. A growing number of researchers underline the impact of nutrition knowledge on diet-related behaviors [8,9,10]. Nutrition knowledge refers to awareness of practices and concepts related to nutrition and health such as adequate food intake, diet-related diseases, foods representing major sources of nutrients, as well as dietary guidelines and recommendations [8,9,10]. The Internet, family members, and friends, as well as television, are the most common sources of nutrition knowledge reported globally [11,12].

In developed countries, a growing number of individuals use individual nutrition plans developed by a dietitian or nutritionist [13]. A dietetic consultation supports individual patients to modify their dietary behaviors to improve health outcomes [14,15]. Dietetic consultations are offered as part of the therapeutic process (e.g., for diabetic patients), but also on a commercial basis (e.g., for those who want to lead a healthy lifestyle) [1,2,16]. A modification of dietary behaviors is crucial for preserving cardiovascular health and effective management of type 2 diabetes [15,16]. As a result of the COVID-19 pandemic, virtual dietetic consultations are gaining popularity [17].

Due to the high global burden of diet-related diseases, numerous public health actions were launched to promote a healthy diet and improve the level of nutrition knowledge [18,19,20]. In numerous countries, food labeling systems with nutrition information were introduced to promote pro-healthy food choices and dietary behaviors [19,20]. Food labeling systems differ across the countries, but two major groups can be identified: numeric data on specific nutrient content and summary labels (e.g., graphic, or color-coded logo) with synthesized information on ingredient and nutrient content [20,21].

The World Health Organization (WHO) encourages member states to introduce front-of-package labels with information about the nutritional value of the food product [22]. In the European Union (EU), there is a law on mandatory nutrition declaration [21,23]. In line with the EU regulations, most pre-packed foods in the EU should provide nutrition information on the energy value of the products as well as the amounts of fat, saturates, carbohydrates, sugars, protein, and salt in the food [23]. However, in most cases, this mandatory nutrition declaration is provided on the back of food packaging [23]. Food labels were proven as a cost-effective and relatively easy-to-follow method of communicating nutrition information to consumers [8]. However, the percentage of consumers who read the food labels during shopping is relatively low [24].

Poland is an EU member with more than 38 million inhabitants. The Polish agri-food sector is one of the biggest in Europe with approximately 3 million people employed in sectors of agriculture, forestry, and food production [25]. Despite the importance of the food industry in the Polish economy, a markable percentage of adults in Poland present a moderate or low level of knowledge about food, nutrition, and their relation to health [12,26]. Moreover, the prevalence of overweight or obesity in Poland is one of the highest in the EU [27,28]. Rapid socioeconomic changes observed in recent decades have affected the lifestyle of Poles, including dietary behaviors [28,29]. The COVID-19 pandemic and lockdown also affected eating behaviors and dietary habits among adults in Poland [30,31]. Regular monitoring of nutrition knowledge and dietary habits is a key element of evidence-based public health policy planning. However, there is limited up-to-date research on diet-related behaviors among adults in Poland, that was carried out after the onset of the pandemic.

This study aimed to assess nutrition knowledge, dietary habits, and food label use among adults in Poland as well as to identify factors associated with diet-related behaviors.

## 2. Materials and Methods

### 2.1. Study Design

This is a cross-sectional survey that was carried out between 1 and 4 July 2022 among adults in Poland. Data was collected by a specialized survey company through computer-assisted web interviews (CAWI method) [32]. Data were collected using a dedicated IT that has been described in previously published papers [18,33]. The study protocol was reviewed and approved by the Ethical Review Board at the Medical University of Warsaw, Poland (number AKBE/176/2022 as of 13 June 2022). Participation in the study was voluntary and anonymous. Informed consent was collected from all the participants.

### 2.2. Study Population

A non-probability quota-based sample of 1070 adult citizens of Poland was selected from the 110,000 registered and verified individual users of the web panel provider (survey company) called the Nationwide Research Panel Ariadna [32]. The stratification model included gender, age, and place of residence. The stratification was based on national demographic data regularly published by the Central Statistical Office of the Republic of Poland.

### 2.3. Measures

The study questionnaire was prepared by the authors for the purpose of this study. The questionnaire included 20 questions on nutrition knowledge, dietary habits, diet-related diseases, and lifestyle. Moreover, a set of questions on sociodemographic characteristics was prepared.

*Dietary habits*: Respondents were asked about their dietary habits, using six questions: “Are you currently on a diet or adhere to certain dietary rules? (yes/no)”; “Have you been on a box or dietary catering diet in the last 12 months? (yes/no); “How many meals do you eat per day?”; “Have you consulted your diet with a doctor or nutritionist in the last 12 months? (yes/no)”; and “Has your doctor ever advised you to change your eating habits or diet because of your health status? (yes, in the last 12 months; yes, over 12 months ago; no)”.

*Nutrition knowledge*: Respondents were asked about the self-reported level of nutrition knowledge, using the question: How would you rate your level of nutrition knowledge? with five possible answers: very good; rather good; moderate; rather bad; very bad.” Moreover, respondents were asked about the sources of nutrition information, using the following question: “What sources do you use to seek information on nutrition and diet? (1) family and friends; (2) physician, nutritionist, or qualified personal trainer; (3) press; (4) radio; (5) television; (6) news websites; (7) blogs, forums, discussion groups; (8) social media, e.g., Facebook, Instagram, TikTok; (9) influencers on Instagram, Facebook, YouTube, etc.; (10) other sources”.

*Public attitudes towards the nutrition labeling information*: Respondents were asked about their attitudes towards the nutrition labeling information, using the questions: “In the last 30 days, have you checked the food labels/nutrition labeling information on the packaging of store-bought meals or food products? (yes/no)” and “Did you check the nutrition information on restaurant menus during your last visit to a fast-food bar or restaurant? (yes/no)”.

### 2.4. Statistical Analysis

All the statistical analyses were carried out with SPSS v. 28 (IBM Corporation, Armonk, NY, USA). The distribution of categorical variables was shown by frequencies and proportions. The chi-square test was used to compare categorical variables.

The logistic regression analyses were used to assess the associations between personal characteristics ((1) gender, (2) age group, (3) having higher education, (4) marital status, (5) having children, (6) number of household members; (7) children in the home; (8) place of residence; (9) occupational status; (10) economic status; (11) presence of chronic diseases; (12) self-reported health status; (13) physical activity; (14) tobacco use; and (15) alcohol consumption) and dietary habits. The following habits: (1) being on a diet; (2) dietary consultation in the last 12 months; (3) dietary change advice by a doctor (ever); (4) eating at least three meals per day; (5) food labels/nutrition labeling information check in the last 30 days; (6) nutrition information on restaurant menus check during the last visit were considered separately as a dependent variable in the model. Data on personal characteristics were considered independent variables. In simple logistic regression analyses, all variables were considered separately. Multivariable logistic regression analyses included all the statistically significant variables in simple models.

The strength of association was measured by the odds ratio (OR) with 95% confidence intervals (95%CI). Statistical inference was based on the criterion *p* < 0.05.

## 3. Results

Completed questionnaires were received from 1070 adult inhabitants of Poland, and 53.3% were females (Table 1). The characteristics of the study population are presented in Table 1.

### 3.1. Sources of Nutrition Knowledge and Dietary Habits

Most of the respondents (52.6%) declared a moderate level of nutrition knowledge and over one quarter (28.1%) of respondents declared a rather good or very good level of nutrition knowledge (Table 2). Among adults in Poland, the most common sources of nutrition knowledge were news websites (41.8%) or family/friends (32.4%). Less than one-quarter of respondents (22.7%) indicated a physician, nutritionist, or qualified personal trainer as a source of nutrition knowledge (Table 2). Over one-fifth of respondents indicated social media as a source of nutrition knowledge (21.6%), and 27.5% of respondents followed healthy eating channels on YouTube (Table 2).

Over one-quarter of adults in Poland were on a diet (28.7%). Out of all respondents (n = 1070), 10.9% were on a slimming diet and 6.4% were on a pro-healthy diet, e.g., gluten-free, low-protein, and low-carbohydrate (Table 2). Among the respondents, 4% were on a box or dietary catering diet in the last 12 months. Over one-tenth of respondents (11.9%) consumed less than three meals per day. Dietary/nutritional consultation with a doctor or nutritionist in the last 12 months was declared by 13.3% of respondents, and 4.6% had dietary/nutritional teleconsultation with a doctor or nutritionist in the last 12 months (Table 2). Almost one-third of respondents (32.6%) were encouraged by a doctor to change their eating habits or diet because of their health status. Half of the respondents (50.3%) declared that they checked food labels/nutrition labeling information on store-bought meals or food products, and 15.4% checked the nutrition information on restaurant menus during their last visit to a fast-food bar or restaurant (Table 2).

### 3.2. Sociodemographic Differences in Nutrition Knowledge and Dietary Habits

Females, respondents with higher education, those who have children, respondents with chronic diseases, as well as those with regular physical activity more often declared (*p* < 0.05) being on diet (Table 3). The prevalence of dietary/nutritional consultation in the last 12 months was significantly higher among respondents with chronic diseases, those from rural areas or the largest cities, and those with regular physical activity (*p* < 0.05). Respondents aged 50 years and over, those who had children, citizens of the largest cities, currently unemployed/retired respondents, as well as those with chronic diseases more often declared (*p* < 0.05) that doctors encouraged them to change dietary habits (Table 3). The prevalence of use of box or dietary catering was the highest among respondents aged 40–49 years, those without children, currently employed/self-employed respondents, those with good financial status as well as those who regularly used alcohol (Table 3). Males, respondents who lived alone, those who did not have children, respondents with bad economic status, those with bad health status, as well as daily smokers more often declared that they consumed less than three meals per day (Table 3). There were also significant sociodemographic differences in the prevalence of respondents who checked food labels/nutrition labeling when shopping or when visiting a restaurant (*p* < 0.05). Details are presented in Table 3.

### 3.3. Factors Associated with Nutrition Knowledge and Dietary Habits

In multivariable logistic regression models (Table 4), female gender (OR:1.70; 95%CI:1.26–2.29; *p* < 0.001), presence of chronic diseases (OR:1.83; 95%CI:1.37–2.44; *p* < 0.001), regular physical activity (*p* < 0.001), being a non-smoker (OR:1.45; 95%CI:1.02–2.06; *p* = 0.04) were significantly associated with higher odds of being on a diet. Presence of chronic diseases (OR:3.15; 95%CI:2.14–4.62; *p* < 0.001) and regular physical activity (*p* < 0.05) were significantly associated with higher odds of dietary consultation in the last 12 months (Table 4). Respondents who lived in cities above 500,000 residents (OR:1.89; 95%CI:1.22–2.94; *p* = 0.005), those with chronic diseases (OR:3.38; 95%CI:2.47–4.63; *p* < 0.001), respondents who declared bad health status (OR:2.21; 95%CI:1.29–3.77; *p* = 0.004), as well as those who undertook physical activity 3–4 times per week (OR:1.83; 95CI: 1.14–2.95; *p* = 0.01) or once per month (OR:2.12; 95%CI:1.01–4.43; *p* = 0.04) had higher odds of being encouraged by a doctor to change their diet.

Respondents who lived alone (OR:1.89; 95%CI:1.16–3.09; *p* = 0.01), those with bad economic status (OR:1.88; 95%CI:1.12–3.13; *p* = 0.02), those with bad health status (OR:2.34; 95%CI:1.28–4.29; *p* = 0.006), as well as daily smokers (OR:2.00; 95%CI:1.33–2.99; *p* < 0.001) had higher odds to consume less than three meals per day (Table 4).

Females (OR:1.63; 95%CI:1.24–2.15; *p* < 0.001), respondents with higher education (OR:1.53; 95%CI:1.17–2.01; *p* = 0.002), those who had never been married (OR:1.49; 95%CI:1.07–2.07; *p* = 0.02), respondents with chronic diseases (OR:1.73; 95%CI:1.30–2.31; *p* < 0.001), those with regular physical activity (*p* < 0.05), as well as non-smokers (OR:1.42; 95%CI:1.04–1.95; *p* = 0.03) had higher odds of checking the food labels/nutrition labeling when shopping. Respondents who lived with children (OR:1.57; 95%CI:1.05–2.35; *p* = 0.03) and those with regular physical activity (*p* < 0.05) had higher odds of checking the nutrition information on restaurant menus (Table 4).

## 4. Discussion

This study produced data on several diet-related behaviors among adults in Poland. In this study, a markable percentage of adults in Poland indicated the Internet (including news websites and social media) as a leading source of nutrition knowledge. A relatively high percentage of adults in Poland (28.7%) followed the diet which may suggest that a growing number of Poles pay attention to what they eat. Despite the growing availability of dietary/nutritional consultation, both in primary care and telemedicine settings, only 13.3% of adults consulted their diet with a doctor or nutritionist. Findings from this study also point to an urgent need to promote the use of food labels, especially among respondents without higher education. The presence of chronic disease and regular physical activity were the most important factors associated with healthy dietary behaviors.

Promoting healthy diets is one of the most common public health interventions worldwide [6]. Educational campaigns, dietary interventions in healthcare settings, and legal actions that support healthy choices and lifestyle changes are the basic tools to increase nutrition knowledge [6,19,20]. Findings from this study showed that only 28.1% of adults in Poland had a good level of nutrition knowledge. Moreover, a markable percentage of adults in Poland indicated the Internet (including news websites and social media) as a major source of nutrition knowledge. This finding is in line with the previously published studies [11,12]. Social media are considered a promising feature for nutrition interventions, especially for adolescents and young adults [34]. However, there are serious concerns about the credibility score of nutritional information on the Internet [35]. The implementation of online medical content validation standards or the promotion of official websites of public health organizations that publish dietary recommendations should be considered for providing evidence-based nutrition knowledge. Moreover, the use of nutrition-related mobile applications may also contribute to the increase of public nutrition knowledge [18].

Findings from this study showed that over one-quarter of adults in Poland followed the diet, wherein a slimming diet was the most common type of diet. This finding suggests that major motivations for being on diet were lifestyle factors/beauty-related factors, rather than health reasons. In this study, females were more likely to be on a diet that is in line with previously published data on gender differences in dietary intakes [36]. We can hypothesize that beauty-related factors and willingness to lose weight before the summer season was the most important reasons for females that declared being on a diet. Non-smokers as well as those with regular physical activity were more likely to follow the diet. Diet is an important lifestyle factor that affects the risk of diseases [6], so respondents who follow the diet also pay attention to other lifestyle factors such as regular physical activity and substance use.

In this study, respondents with chronic diseases were more likely to follow the diet, had a consultation with a doctor or nutritionist, and were encouraged by a doctor to change their diet. Dietary change advice after the diagnosis of chronic diseases is one of the best practices to reduce burdens of diet-related (e.g., cardiovascular diseases or diabetes) [1]. However, in this study, the percentage of respondents with chronic diseases who followed a diet or used dietary consultation was relatively low. Dietary guidelines for the prevention and management of chronic diseases differ by the location of the disease [37]. Dietary interventions offered to patients with chronic diseases should be tailored to the individual nutrition and health literacy level [38]. Diet-related mobile applications may significantly improve the dietary habits of patients with chronic diseases and provide nutrition information in an understandable way [18].

Respondents who lived alone, smokers, respondents with bad health status as well as those with bad economic status were more likely to eat less than three meals per day. Most of the dietary recommendations in Poland recommend at least three meals per day (breakfast, dinner, and supper) [39]. We can hypothesize that low socioeconomic status and bad financial situation are the most critical factors determining the consumption of fewer than three meals per day. Findings from this study may indicate food insecurity in some populations in Poland, especially older Poles who lived alone [40].

The introduction of food labeling systems is one of the most effective public health interventions to promote pro-healthy food choices and dietary behaviors. However, numerous studies reported difficulties in consumers’ understanding of food labels [19,20,21]. Moreover, a variety of food label systems (e.g., differences in the Nutri score implementation in the EU) can also influence the use of food labels by consumers in different countries [19,20]. Moreover, nutrition literacy level may also influence public attitudes toward the use of food labels [30]. In this study, females were more likely to use food labels, which may result from the fact that in Poland females are more often responsible for food preparation at home. Moreover, respondents with higher education were more likely to use food labels. Education is one of the most important socioeconomic factors associated with health that was described in numerous studies [41]. Respondents with chronic diseases were more likely to use food labels that may result from dietary change advice that is a part of disease management recommendations for the patients. Moreover, the percentage of respondents who used food labels (both when shopping and on the restaurant menus) increased with their physical activity level. This finding confirms the hypothesis that physically active individuals also take care of diet, as diet and physical activity are major lifestyle risk factors for diseases. Moreover, respondents who had children in the home were more likely to check the nutrition information on restaurant menus. We can hypothesize that respondents with children may visit fast-food restaurant chains or restaurants with a kid’s menu, so they pay more attention to the nutrition information on menus.

This study has several practical implications. First, there is a need to promote evidence-based sources of nutrition knowledge among adults in Poland. Organizational and financial barriers to accessing dietary/nutritional consultation with a doctor or nutritionist should be removed. Second, socioeconomic differences in dietary habits presented in this study point to health inequalities. More than one-tenth of respondents consumed less than three meals per day. Further social care activities are needed to support socially disadvantaged groups. Third, educational campaigns on the basic principles of food label use are needed. Moreover, a public-private partnership should be developed to promote food label use in Poland.

Public health authorities should consider introducing legislative interventions to strengthen nutrition knowledge in Poland. The widespread implementation of the Nutri Score system, due to the ease of data interpretation, may contribute to the improvement of nutritional knowledge [19]. Moreover, taxes on unhealthy food should be extended [42]. In 2021, Poland implemented a sugar tax [42]. Preliminary findings showed that the implementation of sugar tax improved population diets [42]. Further actions are needed to extend the list of unhealthy products that are covered by sin taxes. Legislative and organizational activities supporting the possibility of purchasing food from local producers (marketplaces, laws supporting local food producers) should be developed.

This study has some limitations. First, the level of nutrition knowledge was self-declared and based on the questions prepared by the authors. A validated international questionnaire of nutrition knowledge was not used in this study. Second, this study was carried out in July, so the percentage of respondents who followed a slimming diet may be higher than in autumn/winter. Third, this study was carried out on a non-probability quota-based sample of adult Internet users in Poland. Nevertheless, more than 90% of household in Poland have Internet access and sampling methods guarantees the representativeness of the study population. Further studies on nutrition knowledge and food label use in different socioeconomic groups are needed to precisely characterize diet-related behaviors, especially among socially disadvantaged groups or patients with chronic medical conditions.

## 5. Conclusions

This study showed a significant gap in nutrition knowledge among adults in Poland. Most adults in Poland do not use scientifically verified sources of knowledge about nutrition, such as dietary/nutritional consultation with a doctor or nutritionist. Over every fourth adult followed the diet, which indicates positive changes in dietary habits in Poland. Individuals with chronic diseases more often showed healthy dietary patterns. The presented data emphasize the further need to promote food label use among adults in Poland.

## Figures and Tables

**Table 1:** Characteristics of the study population (n = 1070).

Variable	n	%
Gender		
female	570	53.3
male	500	46.7
Age (years)		
18–29	236	22.1
30–39	214	20.0
40–49	182	17.0
50–59	190	17.8
60+	248	23.2
Educational level		
primary	24	2.2
vocational	107	10.0
secondary	475	44.4
higher	464	43.4
Ever married		
yes	403	37.7
no	667	62.3
Having children		
yes	677	63.3
no	393	36.7
Number of household members		
living alone	147	13.7
living with at least one person	923	86.3
Children under 18 years in home		
yes	372	34.8
no	698	65.2
Place of residence		
rural	357	33.4
city below 20,000 residents	135	12.6
city from 20,000 to 99,999 residents	227	21.2
city from 100,000 to 499,999 residents	202	18.9
city above 500,000 residents	149	13.9
Occupational status		
active	666	62.2
passive	404	37.8
Self-reported economic status		
rather good, good or very good	410	38.3
moderate/difficult to tell	430	40.2
rather bad, bad or very good	230	21.5
Presence of chronic diseases		
yes	481	45.0
no	589	55.0

**Table 2:** Respondents’ knowledge regarding nutrition and dietary habits (n = 1070).

Variable	n	%
Self-reported level of nutrition knowledge		
very good	67	6.3
rather good	233	21.8
moderate	563	52.6
rather bad	146	13.6
very bad	61	5.7
Sources of nutrition knowledge		
family and friends	347	32.4
physician, nutritionist, or qualified personal trainer	243	22.7
press	155	14.5
radio	90	8.4
television	276	25.8
news websites	447	41.8
blogs, Internet forums, discussion groups	213	19.9
social media, e.g., Facebook, Instagram, TikTok	231	21.6
YouTube, e.g., healthy eating channels	294	27.5
influencers on Instagram, Facebook, YouTube, etc.	110	10.3
other sources of information	83	7.9
Being on a diet		
yes	307	28.7
no	763	71.3
Type of diet		
slimming diet	117	10.9
pro-healthy diet (e.g., gluten-free; low-protein; low-carbohydrate)	69	6.4
vegetarian	30	2.8
vegan	5	0.5
other types of diet	86	8.0
Being on a box or dietary catering diet in the last 12 months		
yes	43	4.0
no	1027	96.0
Number of meals per day		
1	13	1.2
2	115	10.7
3	500	46.7
4	313	29.3
5 or more	129	12.1
Dietary/nutritional consultation with a doctor or nutritionist in the last 12 months		
yes	142	13.3
no	928	86.7
Dietary/nutritional teleconsultation with a doctor or nutritionist in the last 12 months		
yes	49	4.6
no	1021	95.4
Has your doctor ever advised you to change your eating habits or diet because of your health status?		
yes, in the last 12 months	103	9.6
yes, over 12 months ago	246	23.0
no	721	67.4
In the last 30 days, have you checked the food labels/nutrition labeling information on the packaging of store-bought meals or food products?		
yes	538	50.3
no	532	49.7
Did you check the nutrition information on restaurant menus during your last visit to a fast-food bar or restaurant?		
yes	165	15.4
no	905	84.6

**Table 3:** Respondents’ dietary habits by sociodemographic and lifestyle variables (n = 1070).

(a)								
Dietary Habits–the Percentage of Respondents Who Answered “Yes” by Sociodemographic Factors								
Variable	Being on a Diet		Dietary/Nutritional Consultation in the Last 12 Months		Dietary/Nutritional Changes Advice by a Doctor (Ever)		Box or Dietary Catering Diet in the Last 12 Months	
	n (%)	*p*	n (%)	*p*	n (%)	*p*	n (%)	*p*
Gender								
female	193 (33.9)	<0.001	85 (14.9)	0.09	180 (31.6)	0.4	22 (3.9)	0.8
male	114 (22.8)		57 (11.4)		169 (33.8)		21 (4.2)	
Age (years)								
18–29	61 (25.8)	0.5	36 (15.3)	0.2	56 (23.7)	<0.001	16 (6.8)	0.004
30–39	64 (29.9)		34 (15.9)		64 (29.9)		6 (2.8)	
40–49	48 (26.4)		15 (8.2)		51 (28.0)		13 (7.1)	
50–59	53 (27.9)		22 (11.6)		77 (40.5)		4 (2.1)	
60+	81 (32.7)		35 (14.1)		101 (40.7)		4 (1.6)	
Educational level								
primary	5 (20.8)	0.006	3 (12.5)	0.1	10 (41.7)	0.4	0 (0.0)	0.07
vocational	16 (15.0)		6 (5.6)		28 (26.2)		1 (0.9)	
secondary	140 (29.5)		67 (14.1)		158 (33.3)		16 (3.4)	
higher	146 (31.5)		66 (14.2)		153 (33.0)		26 (5.6)	
Ever married								
yes	195 (29.2)	0.6	90 (13.5)	0.8	234 (35.1)	0.03	24 (3.6)	0.4
no	112 (27.8)		52 (12.9)		190 (28.5)		19 (4.7)	
Having children								
yes	212 (31.3)	0.01	90 (13.3)	0.9	241 (35.6)	0.006	20 (3.0)	0.02
no	95 (24.2)		52 (13.2)		108 (27.5)		23 (5.9)	
Number of household members								
living alone	43 (29.3)	0.9	21 (14.3)	0.7	44 (29.9)	0.5	7 (4.8)	0.6
living with at least one person	264 (28.6)		121 (13.1)		305 (33.0)		36 (3.9)	
Children under 18 years in home								
yes	110 (29.6)	0.6	51 (13.7)	0.8	108 (29.0)	0.07	17 (4.6)	0.5
no	197 (28.2)		91 (13.0)		241 (34.5)		26 (3.7)	
Place of residence								
rural	94 (26.3)	0.5	47 (13.2)	0.049	96 (26.9)	0.01	8 (2.2)	0.2
city below 20,000 residents	37 (27.4)		13 (9.6)		43 (31.9)		6 (4.4)	
city from 20,000 to 99,999 residents	75 (33.0)		38 (16.7)		83 (36.6)		9 (4.0)	
city from 100,000 to 499,999 residents	60 (29.7)		18 (8.9)		65 (32.2)		10 (5.0)	
city above 500,000 residents	41 (27.5)		26 (17.4)		62 (41.6)		10 (6.7)	
Occupational status								
active	179 (26.9)	0.09	84 (12.6)	0.4	197 (29.6)	0.007	36 (5.4)	0.003
passive	128 (31.7)		58 (14.4)		152 (37.6)		7 (1.7)	
Self-reported economic status								
rather good, good or very good	125 (30.5)	0.6	47 (11.5)	0.1	121 (29.5)	0.04	24 (5.9)	0.03
moderate/difficult to tell	117 (27.2)		56 (13.0)		138 (32.1)		10 (2.3)	
rather bad, bad or very good	65 (28.3)		39 (17.0)		90 (39.1)		9 (3.9)	
Presence of chronic diseases								
yes	168 (34.9)	<0.001	96 (20.0)	<0.001	237 (49.3)	<0.001	19 (4.0)	0.9
no	139 (23.6)		46 (7.8)		112 (19.0)		24 (4.1)	
Self-reported health status								
rather good, good or very good	139 (29.4)	0.9	54 (11.4)	0.2	111 (23.5)	<0.001	23 (4.9)	0.4
moderate/difficult to tell	141 (28.1)		72 (14.3)		184 (36.7)		16 (3.2)	
rather bad, bad or very good	27 (28.1)		16 (16.7)		54 (56.3)		4 (4.2)	
Physical activity								
everyday	72 (40.9)	<0.001	43 (24.4)	<0.001	59 (33.5)	0.6	12 (6.8)	0.2
3–4 times per week	82 (42.5)		32 (16.6)		65 (33.7)		9 (4.7)	
1–2 times per week	69 (31.4)		22 (10.0)		72 (32.7)		9 (4.1)	
2–3 times per month	25 (25.5)		15 (15.3)		33 (33.7)		3 (3.1)	
once per month	9 (20.9)		4 (9.3)		19 (44.2)		3 (7.0)	
less than once per month	20 (14.0)		18 (12.6)		46 (32.2)		4 (2.8)	
never	30 (15.2)		8 (4.1)		55 (27.9)		3 (1.5)	
Daily smoking								
yes	57 (22.3)	0.009	26 (10.2)	0.09	86 (33.6)	0.7	13 (5.1)	0.3
no	250 (30.7)		116 (14.3)		263 (32.3)		30 (3.7)	
Alcohol consumption								
everyday	17 (33.3)	0.2	8 (15.7)	0.8	18 (35.3)	0.6	4 (7.8)	0.007
3–4 times per week	26 (23.6)		13 (11.8)		40 (36.4)		4 (3.6)	
1–2 times per week	64 (27.2)		27 (11.5)		67 (28.5)		19 (8.1)	
2–3 times per month	45 (24.2)		24 (12.9)		61 (32.8)		3 (1.6)	
once per month	30 (25.9)		14 (12.1)		34 (29.3)		2 (1.7)	
less than once per month	71 (33.0)		30 (14.0)		77 (35.8)		6 (2.8)	
never	54 (34.4)		26 (16.6)		52 (33.1)		5 (3.2)	
**(b)**								
**Variable**	**Eating Less Than 3 Meals per Day**			**Checking the Food Labels/Nutrition Labeling When Shopping**			**Checking the Nutrition Information on Restaurant Menus**	
	**n** **(%)**	***p***		**n** **(%)**	***p***		**n** **(%)**	***p***
Gender								
female	57 (10.0)	0.04		316 (55.4)	<0.001		95 (16.7)	0.2
male	71 (14.2)			222 (44.4)			70 (14.0)	
Age (years)								
18–29	26 (11.0)	0.9		129 (54.7)	0.04		48 (20.3)	0.05
30–39	24 (11.2)			120 (56.1)			37 (17.3)	
40–49	24 (13.2)			92 (50.5)			28 (15.4)	
50–59	24 (12.6)			81 (42.6)			21 (11.1)	
60+	30 (12.1)			116 (46.8)			31 (12.5)	
Educational level								
primary	2 (8.3)	0.3		9 (37.5)	<0.001		5 (20.8)	0.1
vocational	16 (15.0)			35 (32.7)			8 (7.5)	
secondary	64 (13.5)			225 (47.4)			76 (16.0)	
higher	46 (9.9)			269 (58.0)			76 (16.4)	
Ever married								
yes	71 (10.6)	0.09		315 (47.2)	0.01		91 (13.6)	0.04
no	57 (14.1)			223 (55.3)			74 (18.4)	
Having children								
yes	71 (10.5)	0.05		330 (48.7)	0.2		95 (14.0)	0.1
no	57 (14.5)			208 (52.9)			70 (17.8)	
Number of household members								
living alone	31 (21.1)	<0.001		68 (46.3)	0.3		24 (16.3)	0.7
living with at least one person	97 (10.5)			470 (50.9)			141 (15.3)	
Children under 18 years in home								
yes	33 (8.9)	0.02		203 (54.6)	0.04		70 (18.8)	0.03
no	95 (13.6)			335 (48.0)			95 (13.6)	
Place of residence								
rural	43 (12.0)	0.8		163 (45.7)	0.2		45 (12.6)	0.5
city below 20,000 residents	13 (9.6)			71 (52.6)			23 (17.0)	
city from 20,000 to 99,999 residents	31 (13.7)			116 (51.1)			39 (17.2)	
city from 100,000 to 499,999 residents	25 (12.4)			104 (51.5)			34 (16.8)	
city above 500,000 residents	16 (10.7)			84 (56.4)			24 (16.1)	
Occupational status								
active	76 (11.4)	0.5		349 (52.4)	0.08		103 (15.5)	0.9
passive	52 (12.9)			189 (46.8)			62 (15.3)	
Self-reported economic status								
rather good, good or very good	37 (9.0)	<0.001		212 (51.7)	0.6		67 (16.3)	0.3
moderate/difficult to tell	45 (10.5)			217 (50.5)			70 (16.3)	
rather bad, bad or very good	46 (20.0)			109 (47.4)			28 (12.2)	
Presence of chronic diseases								
yes	57 (11.9)	0.9		259 (53.8)	0.04		82 (17.0)	0.2
no	71 (12.1)			279 (47.4)			83 (14.1)	
Self-reported health status								
rather good, good or very good	42 (8.9)	<0.001		253 (53.6)	0.05		87 (18.4)	0.048
moderate/difficult to tell	62 (12.4)			246 (49.0)			67 (13.3)	
rather bad, bad or very good	24 (25.0)			39 (40.6)			11 (11.5)	
Physical activity								
everyday	18 (10.2)	0.4		103 (58.5)	<0.001		43 (24.4)	<0.001
3–4 times per week	22 (11.4)			127 (65.8)			47 (24.4)	
1–2 times per week	20 (9.1)			130 (59.1)			35 (15.9)	
2–3 times per month	13 (13.3)			50 (51.0)			16 (16.3)	
once per month	8 (18.6)			20 (46.5)			1 (2.3)	
less than once per month	17 (11.9)			61 (42.7)			10 (7.0)	
never	30 (15.2)			47 (23.9)			13 (6.6)	
Daily smoking								
yes	47 (18.4)	<0.001		104 (40.6)	<0.001		34 (13.3)	0.3
no	81 (10.0)			434 (53.3)			131 (16.1)	
Alcohol consumption								
everyday	10 (19.6)	0.1		21 (41.2)	0.2		11 (21.6)	0.9
3–4 times per week	19 (17.3)			51 (46.4)			17 (15.5)	
1–2 times per week	25 (10.6)			131 (55.7)			33 (14.0)	
2–3 times per month	16 (8.6)			98 (52.7)			28 (15.1)	
once per month	11 (9.5)			56 (48.3)			20 (17.2)	
less than once per month	25 (11.6)			111 (51.6)			33 (15.3)	
never	22 (14.0)			70 (44.6)			23 (14.6)	

**Table 4:** Factors associated with dietary habits among adults in Poland (n = 1070).

(a)												
Factors Associated with Dietary Habits among Adults in Poland												
Variable	Being on a Diet				Dietary/Nutritional Consultation in the Last 12 Months				Dietary/Nutritional Changes Advice by a Doctor (Ever)			
	Univariate Logistic Regression		Multivariable Logistic Regression		Univariate Logistic Regression		Multivariable Logistic Regression		Univariate Logistic Regression		Multivariable Logistic Regression	
	*p*	OR (95%CI)	*p*	OR (95%CI)	*p*	OR (95%CI)	*p*	OR (95%CI)	*p*	OR (95%CI)	*p*	OR (95%CI)
Gender												
female	<0.001	1.73 (1.32–2.27)	<0.001	1.70 (1.26–2.29)	0.09	1.36 (0.95–1.95)			0.4	0.90 (0.70–1.17)		
male		Reference		Reference		Reference				Reference		
Age (years)												
18–29		Reference			0.7	1.10 (0.66–1.81)				Reference		Reference
30–39	0.3	1.39 (0.94–2.06)			0.6	1.15 (0.69–1.92)			0.1	1.37 (0.90–2.09)	0.3	1.29 (0.80–2.06)
40–49	0.6	1.11 (0.72–1.71)			0.06	0.55 (0.29–1.03)			0.3	1.25 (0.81–1.95)	0.9	0.97 (0.58–1.63)
50–59	0.9	1.03 (0.66–1.60)			0.4	0.80 (0.45–1.41)			<0.001	2.19 (1.44–3.32)	0.4	1.26 (0.76–2.10)
60+	0.3	1.22 (0.81–1.85)				Reference			<0.001	2.21 (1.49–3.27)	0.9	1.04 (0.60–1.78)
Having higher education												
yes	0.08	1.27 (0.97–1.66)			0.4	1.16 (0.81–1.65)			0.8	1.03 (0.80–1.33)		
no		Reference				Reference				Reference		
Ever married												
yes	0.6	1.07 (0.82–1.41)			0.8	1.05 (0.73–1.52)			0.03	1.35 (1.04–1.77)	0.9	1.04 (0.71–1.52)
no		Reference				Reference				Reference		Reference
Having children												
yes	0.01	1.43 (1.08–1.90)	0.08	1.32 (0.97–1.79)	0.9	1.01 (0.70–1.45)			0.006	1.46 (1.11–1.91)	0.4	1.18 (0.80–1.74)
no		Reference		Reference		Reference				Reference		Reference
Number of household members												
living alone	0.9	1.03 (0.70–1.51)			0.7	1.11 (0.67–1.82)			0.5	0.87 (0.59–1.26)		
living with at least one person		Reference				Reference				Reference		
Children under 18 years in home												
yes	0.6	1.07 (0.81–1.41)			0.8	1.06 (0.73–1.53)			0.07	0.78 (0.59–1.02)		
no		Reference				Reference				Reference		
Place of residence												
rural	0.8	0.94 (0.61–1.45)				Reference				Reference		Reference
city below 20,000 residents	0.9	1.00 (0.59–1.68)			0.3	0.70 (0.37–1.35)			0.3	1.27 (0.83–1.96)	0.3	1.31 (0.82–2.10)
city from 20,000 to 99,999 residents	0.3	1.30 (0.83–2.05)			0.2	1.33 (0.83–2.11)			0.01	1.57 (1.10–2.24)	0.1	1.39 (0.94–2.04)
city from 100,000 to 499,999 residents	0.7	1.11 (0.70–1.78)			0.1	0.65 (0.36–1.14)			0.2	1.29 (0.89–1.88)	0.1	1.37 (0.91–2.06)
city above 500,000 residents		Reference			0.2	1.39 (0.83–2.35)			0.001	1.94 (1.30–2.89)	0.005	1.89 (1.22–2.94)
Occupational status												
active		Reference			0.4	0.86 (0.60–1.23)			0.007	1.44 (1.11–1.87)	0.9	0.97 (0.69–1.37)
passive	0.09	1.26 (0.96–1.65)				Reference				Reference		Reference
Self-reported economic status												
rather good, good or very good	0.6	1.11 (0.78–1.59)			0.05	0.63 (0.40–1.00)				Reference		Reference
moderate/difficult to tell	0.8	0.95 (0.66–1.36)			0.17	0.73 (0.47–1.14)			0.07	1.36 (0.98–1.90)	0.8	0.97 (0.70–1.33)
rather bad, bad or very good		Reference				Reference			0.01	1.54 (1.09–2.16)	0.7	1.07 (0.72–1.57)
Presence of chronic diseases												
yes	<0.001	1.74 (1.33–2.27)	<0.001	1.83 (1.37–2.44)	<0.001	2.94 (2.02–4.28)	<0.001	3.15 (2.14–4.62)	<0.001	4.14 (3.15–5.43)	<0.001	3.38 (2.47–4.63)
no		Reference		Reference		Reference		Reference		Reference		Reference
Self-reported health status												
rather good, good or very good	0.8	1.07 (0.66–1.74)			0.2	0.65 (0.35–1.19)				Reference		Reference
moderate/difficult to tell	0.9	1.00 (0.61–1.62)			0.6	0.84 (0.46–1.51)			<0.001	2.22 (1.43–3.46)	0.08	1.34 (0.97–1.85)
rather bad, bad or very good		Reference				Reference			<0.001	4.18 (2.65–6.60)	0.004	2.21 (1.29–3.77)
Physical activity												
everyday	<0.001	3.85 (2.36–6.30)	<0.001	3.87 (2.33–6.42)	<0.001	7.64 (3.48–16.77)	<0.001	8.06 (3.64–17.85)	0.2	1.30 (0.84–2.04)	0.1	1.50 (0.93–2.43)
3–4 times per week	<0.001	4.11 (2.54–6.66)	<0.001	4.79 (2.89–7.95)	<0.001	4.70 (2.10–10.48)	<0.001	5.51 (2.44–12.42)	0.2	1.31 (0.85–2.02)	0.01	1.83 (1.14–2.95)
1–2 times per week	<0.001	2.54 (1.57–4.12)	<0.001	2.95 (1.78–4.88)	0.02	2.63 (1.14–6.04)	0.02	2.80 (1.21–6.49)	0.3	1.26 (0.83–1.91)	0.06	1.54 (0.98–2.44)
2–3 times per month	0.03	1.91 (1.05–3.47)	0.01	2.23 (1.20–4.16)	0.001	4.27 (1.74–10.46)	<0.001	4.96 (2.00–12.31)	0.3	1.31 (0.78–2.21)	0.06	1.73 (0.98–3.04)
once per month	0.4	1.47 (0.64–3.38)	0.3	1.58 (0.67–3.72)	0.2	2.42 (0.70–8.45)	0.2	2.40 (0.68–8.46)	0.04	2.04 (1.04–4.03)	0.04	2.12 (1.01–4.43)
less than once per month	0.8	1.47 (0.49–1.67)	0.7	0.89 (0.47–1.66)	0.005	3.40 (1.44–8.06)	0.004	3.59 (1.50–8.58)	0.4	1.22 (0.77–1.96)	0.25	1.35 (0.81–2.24)
never		Reference		Reference		Reference		Reference		Reference		Reference
Daily smoking												
yes	0.009	Reference		Reference	0.09	Reference			0.7	1.06 (0.79–1.43)		
no		1.55 (1.11–2.15)	0.04	1.45 (1.02–2.06)		1.47 (0.94–2.31)				Reference		
Alcohol consumption												
everyday	0.9	0.95 (0.49–1.86)	0.4	1.35 (0.65–2.81)	0.9	0.94 (0.40–2.22)			0.8	1.10 (0.57–2.14)		
3–4 times per week	0.06	0.59 (0.34–1.02)	0.2	0.66 (0.36–1.19)	0.3	0.68 (0.33–1.38)			0.6	1.15 (0.69–1.92)		
1–2 times per week	0.1	0.71 (0.46–1.11)	0.1	0.67 (0.41–1.08)	0.2	0.65 (0.37–1.17)			0.3	0.81 (0.52–1.25)		
2–3 times per month	0.04	0.61 (0.38–0.97)	0.009	0.51 (0.30–0.84)	0.3	0.75 (0.41–1.36)			0.9	0.99 (0.63–1.55)		
once per month	0.1	0.67 (0.39–1.13)	0.1	0.66 (0.37–1.16)	0.3	0.69 (0.34–1.39)			0.5	0.84 (0.50–1.41)		
less than once per month	0.8	0.94 (0.61–1.45)	0.4	0.82 (0.51–1.32)	0.5	0.82 (0.46–1.45)			0.6	1.13 (0.73–1.74)		
never		Reference		Reference		Reference				Reference		
**(b)**												
**Factors Associated with Dietary Habits among Adults in Poland**												
**Variable**	**Eating Less Than 3 Meals per Day**				**Checking the Food Labels/Nutrition Labeling** **When Shopping**				**Checking the Nutrition Information on Restaurant Menus**			
	**Univariate** **Logistic** **Regression**		**Multivariable** **Logistic** **Regression**		**Univariate** **Logistic** **Regression**		**Multivariable** **Logistic** **Regression**		**Univariate** **Logistic** **Regression**		**Multivariable** **Logistic** **Regression**	
	***p***	**OR (95%CI)**	***p***	**OR (95%CI)**	***p***	**OR (95%CI)**	***p***	**OR (95%CI)**	***p***	**OR (95%CI)**	***p***	**OR (95%CI)**
Gender												
female		Reference		Reference	<0.001	1.56 (1.22–1.98)	<0.001	1.63 (1.24–2.15)	0.2	1.23 (0.88–1.72)		
male	0.04	1.49 (1.03–2.16)	0.08	1.41 (0.96–2.07)		Reference		Reference		Reference		
Age (years)												
18–29	0.7	0.90 (0.52–1.57)			0.08	1.37 (0.96–1.96)	0.4	1.25 (0.77–2.02)	0.02	1.79 (1.09–2.92)	0.4	1.31 (0.72–2.39)
30–39	0.8	0.92 (0.52–1.63)			0.04	1.45 (1.01–2.10)	0.1	1.44 (0.89–2.32)	0.1	1.46 (0.87–2.45)	0.8	1.10 (0.60–2.01)
40–49	0.7	1.10 (0.62–1.96)			0.3	1.16 (0.79–1.71)	0.3	1.27 (0.79–2.04)	0.4	1.27 (0.73–2.21)	0.9	1.04 (0.56–1.93)
50–59	0.9	1.05 (0.59–1.86)			0.4	0.85 (0.58–1.24)	0.4	0.84 (0.55–1.28)	0.6	0.87 (0.48–1.57)	0.6	0.83 (0.45–1.53)
60+		Reference				Reference		Reference		Reference		Reference
Having higher education												
yes	0.07	0.70 (0.48–1.03)			<0.001	1.73 (1.35–2.21)	0.002	1.53 (1.17–2.01)	0.5	1.14 (0.82–1.59)		
no		Reference				Reference		Reference		Reference		
Ever married												
yes	0.09	0.72 (0.50–1.05)				Reference		Reference		Reference		Reference
no		Reference			0.01	1.38 (1.08–1.78)	0.02	1.49 (1.07–2.07)	0.04	1.42 (1.02–1.99)	0.2	1.37 (0.89–2.09)
Having children												
yes	0.05	0.69 (0.48–1.01)			0.2	0.85 (0.66–1.09)			0.1	0.75 (0.54–1.06)		
no		Reference				Reference				Reference		
Number of household members												
living alone	<0.001	2.28 (1.45–3.56)	0.01	1.89 (1.16–3.09)	0.3	0.83 (0.59–1.18)			0.7	1.08 (0.68–1.74)		
living with at least one person		Reference		Reference		Reference				Reference		
Children under 18 years in home												
yes		Reference		Reference	0.04	1.30 (1.01–1.68)	0.2	1.25 (0.91–1.73)	0.03	1.47 (1.05–2.06)	0.03	1.57 (1.05–2.35)
no	0.02	1.62 (1.07–2.46)	0.2	1.31 (0.84–2.06)		Reference		Reference		Reference		Reference
Place of residence												
rural	0.7	1.14 (0.62–2.09)				Reference		Reference		Reference		
city below 20,000 residents	0.8	0.89 (0.41–1.92)			0.2	1.32 (0.89–1.96)	0.09	1.46 (0.95–2.24)	0.2	1.42 (0.82–2.46)		
city from 20,000 to 99,999 residents	0.4	1.32 (0.69–2.50)			0.2	1.24 (0.89–1.74)	0.2	1.27 (0.88–1.83)	0.1	1.44 (0.90–2.29)		
city from 100,000 to 499,999 residents	0.6	1.17 (0.60–2.29)			0.2	1.26 (0.89–1.79)	0.2	1.28 (0.88–1.87)	0.2	1.40 (0.87–2.28)		
city above 500,000 residents		Reference			0.03	1.54 (1.05–2.26)	0.08	1.48 (0.96–2.28)	0.3	1.33 (0.78–2.28)		
Occupational status												
active	0.5	0.87 (0.60–1.27)			0.08	1.25 (0.98–1.60)			0.9	1.01 (0.72–1.42)		
passive		Reference				Reference				Reference		
Self-reported economic status												
rather good, good or very good		Reference		Reference	0.3	1.19 (0.86–1.64)			0.2	1.41 (0.88–2.26)		
moderate/difficult to tell	0.5	1.18 (0.75–1.86)	0.8	1.06 (0.66–1.71)	0.5	1.13 (0.82–1.56)			0.2	1.40 (0.88–2.25)		
rather bad, bad or very good	<0.001	2.52 (1.58–4.02)	0.02	1.88 (1.12–3.13)		Reference				Reference		
Presence of chronic diseases												
yes	0.9	0.98 (0.68–1.42)			0.04	1.30 (1.02–1.65)	<0.001	1.73 (1.30–2.31)	0.2	1.25 (0.90–1.75)		
no		Reference				Reference		Reference		Reference		
Self-reported health status												
rather good, good or very good		Reference		Reference	0.02	1.69 (1.08–2.64)			0.1	1.75 (0.89–3.41)		
moderate/difficult to tell	0.08	1.44 (0.95–2.18)	0.5	1.18 (0.76–1.82)	0.1	1.40 (0.90–2.19)			0.6	1.19 (0.60–2.35)		
rather bad, bad or very good	<0.001	3.41 (1.95–5.98)	0.006	2.34 (1.28–4.29)		Reference				Reference		
Physical activity												
everyday	0.2	0.63 (0.34–1.18)			<0.001	4.50 (2.89–7.02)	<0.001	4.53 (2.85–7.18)	<0.001	4.58 (2.37–8.85)	<0.001	4.53 (2.33–8.79)
3–4 times per week	0.3	0.72 (0.40–1.29)			<0.001	6.14 (3.95–9.56)	<0.001	5.65 (3.55–9.00)	<0.001	4.56 (2.38–8.74)	<0.001	4.26 (2.21–8.20)
1–2 times per week	0.06	0.56 (0.31–1.02)			<0.001	4.61 (3.02–7.04)	<0.001	4.19 (2.68–6.56)	0.004	2.68 (1.37–5.23)	0.007	2.52 (1.29–4.94)
2–3 times per month	0.7	0.85 (0.42–1.72)			<0.001	3.32 (1.99–5.56)	<0.001	3.05 (1.77–5.26)	0.01	2.76 (1.27–6.01)	0.02	2.56 (1.17–5.61)
once per month	0.6	1.27 (0.54–3.01)			0.003	2.78 (1.40–5.49)	0.02	2.44 (1.19–4.99)	0.3	0.34 (0.04–2.65)	0.3	0.31 (0.04–2.47)
less than once per month	0.4	0.75 (0.40–1.42)			<0.001	2.37 (1.49–3.78)	0.003	2.10 (1.29–3.42)	0.9	1.06 (0.45–2.50)	0.9	0.99 (0.42–2.34)
never		Reference				Reference		Reference		Reference		Reference
Daily smoking												
yes	<0.001	2.04 (1.38–3.01)	<0.001	2.00 (1.33–2.99)		Reference		Reference	0.3	0.80 (0.53–1.20)		
no		Reference		Reference	<0.001	1.67 (1.26–2.22)	0.03	1.42 (1.04–1.95)		Reference		
Alcohol consumption												
everyday	0.2	0.63 (0.34–1.18)			0.7	0.87 (0.46–1.65)	0.6	1.20 (0.60–2.43)	0.2	1.60 (0.72–3.57)		
3–4 times per week	0.3	0.72 (0.40–1.29)			0.8	1.07 (0.66–1.75)	0.4	1.26 (0.73–2.18)	0.9	1.07 (0.54–2.10)		
1–2 times per week	0.06	0.56 (0.31–1.02)			**0.03**	1.57 (1.04–2.35)	0.1	1.45 (0.92–2.27)	0.9	0.95 (0.54–1.69)		
2–3 times per month	0.7	0.85 (0.42–1.72)			0.1	1.38 (0.90–2.12)	0.6	1.13 (0.71–1.81)	0.9	1.03 (0.57–1.88)		
once per month	0.6	1.27 (0.54–3.01)			0.5	1.16 (0.72–1.88)	0.7	1.10 (0.65–1.86)	0.6	1.21 (0.63–2.33)		
less than once per month	0.3	0.75 (0.40–1.42)			0.2	1.33 (0.88–2.01)	0.8	1.07 (0.68–1.69)	0.9	1.06 (0.59–1.88)		
never		Reference				Reference		Reference		Reference		

## Data Availability

Data are available on reasonable request. The dataset used to conduct the analyses is available from the corresponding author on reasonable request.
